# Longitudinal Associations Between Family Socioeconomic Status and Adolescent Depressive Symptom Trajectories in China: A Chain Multiple Mediation Model

**DOI:** 10.1007/s10964-026-02355-4

**Published:** 2026-04-07

**Authors:** Jing Zeng, Mei Zhang, Yafeng Li, Yunting Chen

**Affiliations:** 1 School of Management, Chengdu University of Traditional Chinese Medicine, Chengdu, 611130 Sichuan China; 2 School of Government, Beijing Normal University, Beijing, 100875 China; 3 School of Population and Global Health, The University of Melbourne, Melbourne, 3000 Australia

**Keywords:** Family socioeconomic status, Depressive symptom trajectories, Maternal mental health, Parenting practices, Self-esteem

## Abstract

Although family socioeconomic status (SES) has been widely associated with adolescent depressive symptoms, less is known about how multidimensional SES shapes depressive symptom trajectories through family and individual mechanisms. Guided by the Family Stress Model, this study examined the associations between family SES and adolescents’ depressive symptom trajectories and tested the mediating roles of maternal mental health, parenting practices, and adolescent self-esteem. Data were drawn from six waves of the China Family Panel Studies (2010–2020), including 2,245 children followed from early childhood into adolescence (*M*_*age*_ = 6.45 years, *SD* = 1.12; 54% female). Latent growth modeling was used to estimate the intercept and slope of depressive symptoms, and structural equation modeling was applied to test mediation pathways. Results showed that across all family SES indicators, there was an indirect effect on the intercept of adolescent depressive symptoms through the sequential mediation of maternal mental health, parenting style, and self-esteem. For the slope of depressive symptoms, all SES indicators exerted indirect effects via self-esteem. Additionally, parental occupational status showed an additional indirect effect on the slope through parenting style. Across all models, higher self-esteem predicted lower initial levels of depressive symptoms but was associated with steeper increases in symptoms over time. These findings highlight the dynamic family and individual processes linking socioeconomic disadvantage to adolescent depressive symptom development.

**Supplementary Information:**

The online version contains supplementary material available at 10.1007/s10964-026-02355-4.

## Introduction

Adolescent depression constitutes a major public health concern, with profound consequences for psychosocial functioning, academic achievement, and long-term mental health outcomes (Thapar et al., 2012). Epidemiological evidence consistently indicates that depressive symptoms increase markedly during adolescence and that their developmental trajectories exhibit substantial interindividual heterogeneity (Schubert et al., 2017). These patterns have prompted a growing shift toward longitudinal and developmental perspectives on depression, emphasizing dynamic symptom trajectories rather than static cross-sectional states. A substantial body of research has demonstrated that low family socioeconomic status (SES) is associated with elevated depressive risk and more adverse symptom trajectories among adolescents (Weinberg et al., 2019). However, the influence of SES on mental health is not a simple direct effect; instead, it operates through a series of nested family processes and individual psychological mechanisms (Kirkbride et al., 2024). For example, socioeconomic disadvantage and chronic family stress may undermine parental mental health and reduce positive parenting practices, which in turn compromise adolescents’ psychological resources, such as self-esteem, thereby increasing vulnerability to depression (Masarik & Conger, 2017). Despite considerable progress in identifying pathways linking SES to adolescent mental health, much of the existing literature has either focused on single mediating mechanisms or conceptualized depressive symptom as a static outcome. Consequently, relatively few studies have examined, within an integrated longitudinal framework, how chained mediating processes across parental domains and individual domains jointly account for the role of family SES in shaping adolescents’ depressive symptom trajectories. To address these gaps, the present longitudinal study of Chinese adolescents investigates the association between family SES and trajectories of depressive symptoms, and further examines the mediating roles of parental mental health, parenting behaviors, and adolescents’ self-esteem in this developmental process.

### Family Socioeconomic Status and Adolescent Depressive Symptoms and Trajectories

Family SES is a multidimensional construct. In studies of child and adolescent development and education, family SES is typically measured using parental educational attainment, occupational status, and household income, either separately or as a composite indicator (Zaneva et al., 2024). Family SES represents a key environmental factor underlying individual differences in development and has substantial implications for children’s and adolescents’ subsequent developmental outcomes (Komulainen et al., 2025). Lower family SES is often associated with greater material hardship, and fewer social and psychological resources, which in turn significantly increase the risk of depressive symptoms among adolescents (Sun & Yuan, 2024).

Longitudinal evidence further suggests that family SES is not only associated with higher initial levels of depressive symptoms but also plays a role in shaping their developmental trajectories. For example, early economic hardship and persistent deprivation have been shown to predict elevated levels of internalizing problems during adolescence (Liang et al., 2024). In addition, socioeconomic stress has been linked to an increased likelihood of following unfavorable symptom trajectories, such as moderately increasing or persistently high depressive symptom patterns (Manohar et al., 2025). However, other studies indicate that the effect of SES on the slope of depressive symptom trajectories may be relatively modest, with its influence operating primarily through more proximal risk factors, including parental depression and parenting style (Lepe et al., 2021).

These findings suggest that the association between family SES and adolescent depressive symptom involves both direct pathways and indirect processes that operate through family dynamics and individual psychological mechanisms. Nevertheless, much of the existing literature has focused on single indicators of SES or conceptualized depression as a static outcome. As a result, how multidimensional family SES shapes both the intercept and slope of depressive symptom trajectories through early family psychosocial processes remains insufficiently understood and warrants further investigation.

### Parental Mental Health and Parenting Practices as Mediating Variables

Parental mental health and parenting practices constitute core family processes that are strongly shaped by family SES. Drawing on the Family Stress Model and risk accumulation theory, the economic strain, resource deprivation, and role burdens associated with low SES exert persistent detrimental effects on parental mental health (Scrimin et al., 2022), with especially strong impacts on mothers who typically assume primary caregiving responsibilities (Heintz-Martin et al., 2022). Such psychological distress often manifests in harsher, less responsive, and less warm parenting behaviors (Fonseca et al., 2020). Research has shown that low family income predict higher levels of parental depressive and more negative parenting practices (Cao et al., 2024). These findings suggest that socioeconomic disadvantage first disrupts parents’ own psychological resources and caregiving capacities, thereby altering the emotional climate of the family.

These parent-level factors are key proximal mechanisms influencing adolescent depressive symptom. Parental mental health problems (Clayborne et al., 2021) and less supportive parenting practices (Fitzsimons et al., 2017) have been consistently associated with elevated levels of depressive symptoms among adolescents. Longitudinal studies further demonstrate that parental depression and harsh parenting are not only related to higher initial levels of adolescent depressive symptoms but also predict their increases over time (Manohar et al., 2025). Mediation analyses provide additional evidence that parental depression and adverse parenting practices partially account for the association between family SES and adolescent depression (Zhang & Han, 2021). Together, these findings confirm the central role of parental mental health and parenting behaviors in transmitting the effects of socioeconomic disadvantage on adolescent depressive symptom.

The impact of SES on mental health shows strong gender differences, with women, particularly mothers, demonstrating greater vulnerability (Williams et al., 2013). As primary caregivers within the family, mothers tend to be more sensitive to resource scarcity and environmental instability, and socially prescribed caregiving roles may render them especially susceptible to psychological burden under conditions of economic hardship (Newland et al., 2013). Consequently, the detrimental effects of low SES on family functioning often operate through compromised maternal mental health, which in turn deteriorates the family emotional environment and ultimately affects children’s psychological and emotional well-being. These findings suggest that socioeconomic disadvantage may indirectly influence adolescents’ emotional development by shaping the family emotional climate. Nevertheless, most existing studies have examined these parental processes separately and have rarely linked them to growth parameters of adolescent depressive symptom trajectories.

### Self-Esteem as a Mediating Variable

Self-esteem, defined as an individual’s global evaluation of self-worth, represents one of the most central psychological resources (Muris & Otgaar, 2023). Research indicates that family SES plays a critical role in shaping adolescents’ self-esteem, with lower SES consistently associated with lower perceived self-worth (Barzoki et al., 2025). Socioeconomic disadvantage may constrain adolescents’ opportunities for achievement, social comparison, and perceived control, thereby undermining the development of a positive self-concept (Krauss et al., 2020). Longitudinal evidence further suggests that early family socioeconomic conditions predict trajectories of self-esteem across adolescence, showing the enduring influence of socioeconomic circumstances on adolescents’ internal psychological resources (Trzesniewski et al., 2006).

Low self-esteem is a well-established risk factor for adolescent depression and is closely linked to negative cognitive styles and the persistence of depressive symptoms (Orth et al., 2008). Meta-analytic evidence further shows that low self-esteem prospectively predicts increases in depressive symptoms, supporting its causal primacy in the development of depression (Sowislo & Orth, 2013). Adolescence represents a critical developmental period for self-concept formation, during which individuals are particularly sensitive to self-evaluative processes (Pfeifer & Berkman, 2018). Thus, self-esteem functions as a salient psychological resource that robustly predicts both concurrent levels of depressive symptoms and longer-term adjustment outcomes (Steiger et al., 2014). Longitudinal studies have further shown that self-esteem independently predicts subsequent changes in depressive symptoms even after controlling for baseline levels of depression, suggesting that self-esteem constitutes a stable vulnerability factor that exerts a persistent influence on the trajectory of depressive symptom development (Masselink et al., 2018). Although prior research has demonstrated that self-esteem can mediate the association between adverse family environments and depression (Huang et al., 2022), relatively few studies have examined self-esteem as a mediating pathway linking family SES to longitudinal trajectories of depressive symptoms.

### Parental Processes and Self-Esteem as Sequential Mediating Variables

Beyond functioning as independent mediators between family SES and depressive symptoms, parental processes and self-esteem may operate within a more complex sequential mediation mechanism. Supportive parenting and psychologically healthy parents provide adolescents with emotional security, affirmation, and opportunities for autonomy, all of which are critical for the development of positive self-esteem (Barber et al., 2005). In contrast, parental psychological distress and less responsive parenting practices are associated with lower levels of adolescent self-esteem, which in turn increases vulnerability to depressive symptoms (Yap et al., 2014). This sequential process suggests that family SES may first shape parent-level processes, which subsequently influence adolescents’ self-esteem and ultimately contribute to the development of depressive symptom trajectories. Although the existing literature has not fully established this sequential mediation model, several studies provide empirical support for specific links within the proposed pathway. For example, early family income influences adolescents’ internalizing problems through the mediating roles of parental depression and sensitive parenting (Liang et al., 2024). Similarly, parental depression, parent-child bonding, and child self-esteem mediate the association between family income and child depression, although these mediators operate in parallel rather than in a strictly sequential manner (Cao et al., 2024) simultaneously examined the mediating effects of parental depression, parent-child bonding, and child self-esteem in the association between family income and child depression, although these mediators operated in parallel rather than in a strictly sequential manner. These findings indicate that integrating parental processes and adolescents’ self-system factors within a single model is both theoretically and empirically sound, and they support the value of examining chained mediating mechanisms to better understand how socioeconomic disadvantage becomes embedded in adolescents’ mental health trajectories.

## Current Study

Existing research has established an association between family SES and adolescent depression, and some studies have examined the mediating roles of parental factors or self-esteem. However, how family SES shapes the longitudinal development of depressive symptoms through a sequential process involving parental mental health, parenting practices and adolescent self-esteem is less understood, particularly in Eastern cultural contexts. To address this gap, the present study adopts a longitudinal design to investigate this sequential mechanism. Specifically, latent growth modeling is employed to characterize adolescents’ depressive symptom trajectories over a three period, capturing the intercept (initial level) and slope (rate of change). Structural equation modeling is then used to test a sequential mediation framework linking family SES, parental processes, adolescent self-esteem, and depressive symptom trajectories. Based on the Family Stress Model, the present study proposes the following hypotheses. First, after controlling for baseline covariates, lower family SES at T1 will predict higher initial levels (intercept) and more rapid increases (slope) in depressive symptoms. Second, parental mental health and parenting practices at T3 will operate as parallel mediators between family SES at T1 and the depressive symptom trajectory parameters. Third, adolescent self-esteem at T3 will mediate the association between family SES at T1 and depressive symptom trajectory parameters. Finally, family SES at T1 will indirectly influence depressive symptom trajectory parameters through its effects on parental mental health and parenting practices at T3.

## Methods

### Participants and Procedure

Data for this study were drawn from the China Family Panel Studies (CFPS), a nationally representative longitudinal survey conducted by the Institute of Social Science Survey (ISSS) at Peking University. The CFPS employs a multistage, probability-proportional-to-size sampling design and covers 25 provinces across China, collecting biennial data at the individual, household, and community levels. The baseline survey was initiated in 2010 and identified all household members and their biological or adopted descendants as permanent respondents for long-term follow-up. The present study used six waves of CFPS data collected between 2010 and 2020 to examine how early family socioeconomic conditions shape depressive symptom trajectories across adolescence. To explore the developmental origins of adolescent depression, the analytic sample included children aged 5–8 at baseline (2010), followed prospectively to ages 15–18 (2020). This age range was selected for three key theoretical reasons. First, ages 5–8 represent a critical period for cognitive and emotional development, during which SES effects are already neurally and behaviorally observable. Research has linked family SES to brain structure differences from age 5 and later cognitive-emotional outcomes, making this an ideal starting point to capture early SES initiation effects (McDermott et al., 2019). Second, SES influences are stage-specific. In early childhood, they primarily shape neural foundations for cognitive control and emotion regulation, while in adolescence, they more strongly manifest as depression and anxiety (Isbell et al., 2024). Tracking participants from ages 5–8 to 15–18 thus spans the full window from “early neural shaping” to “adolescent symptom emergence”. Third, this starting point aligns with the Family Stress Model, which posits that economic hardship undermines parental mental health and parenting quality, ultimately affecting child development (Masarik & Conger, 2017). Measuring family SES at ages 5–8 effectively captures its initiating effects on subsequent family processes (e.g., maternal mental health, parenting) and child self-esteem. The final analytic sample included 2,245 participants at baseline (Mage = 6.45 years, SD = 1.12). Among them, 54.25% were male, 36.70% resided in urban areas, and 63.30% in rural areas. Of the participants, 23.12% were only children, and 61.83% lived with both parents at baseline. Family SES was assessed in 2010 (T1). Parenting practices, maternal mental health, and adolescents’ self-esteem were measured in 2014 (T3). Depressive symptoms were assessed at three subsequent waves in 2016, 2018, and 2020 and modeled as latent growth trajectories capturing both initial levels and rates of change across adolescence.​.

### Measures

#### Family SES

Family SES was conceptualized as a multidimensional construct reflecting parental educational attainment, occupational status, and household economic resources (Davis et al., 2010). Consistent with prior research, three indicators measured at baseline were used to represent family SES. First, household economic resources were assessed using annual household income per capita. The CFPS asked respondents who were familiar with household finances to report total household income during the past 12 months, along with household size. Household income per capita was calculated by dividing total household income by household size and was log-transformed to reduce skewness. Second, parental occupational status was measured using the higher of both parents’ scores on the International Socio-Economic Index of Occupational Status (ISEI), a widely used continuous indicator that integrates information on education and income associated with occupations. ISEI scores range from 10 to 90, with higher scores indicating higher occupational standing. In the present sample, the higher parental ISEI score ranged from 19 to 88. Third, parental education level was measured by the higher of both parents’ years of schooling reported in the adult questionnaire, ranging from 0 to 19 years. Each SES indicator was analyzed separately to examine potentially distinct pathways linking family socioeconomic conditions to adolescent mental health outcomes.

#### Depressive Symptoms

Depressive symptoms were assessed using the Center for Epidemiologic Studies Depression Scale (CES-D) in the China Family Panel Studies (CFPS). This scale is a widely used instrument for measuring depressive symptoms in population-based research (Liu et al., 2023). Adolescents’ depressive symptoms were assessed using the 8-item version of the CES-D, a widely used and well-validated self-report measure of depressive symptoms in adolescent populations. To ensure measurement consistency across waves, the same CES-D 8-item version was administered at all three time points. Participants reported the frequency of depressive symptoms experienced during the past week on a 4-point scale ranging from 1 (rarely or none of the time) to 4 (most or all of the time), with higher scores indicating greater depressive symptom severity. Longitudinal measurement invariance of the CES-D scale was tested prior to latent growth modeling. Results supported configural and metric invariance across waves, indicating adequate comparability over time. Internal consistency was acceptable, with Cronbach’s α values of 0.72 (2016), 0.73 (2018), and 0.76 (2020).

#### Maternal Mental Health

Given that mothers often occupy a central role in daily caregiving and emotional interactions within the family, maternal mental health represents a particularly salient aspect of the psychosocial environment of children and adolescents. Research indicates that mothers frequently assume primary caregiving and emotional responsibilities, and that their psychological well-being strongly and directly influences offspring’s emotional development (Fulco et al., 2020). Maternal mental health was assessed using the Kessler Psychological Distress Scale (K6), a widely validated screening instrument for non-specific psychological distress in population-based surveys. Mothers were asked how frequently during the past 30 days they experienced symptoms such as feeling nervous, hopeless, restless or fidgety, so depressed that nothing could cheer them up, that everything was an effort, or worthless. Each item was rated on a 5-point scale ranging from 0 (none of the time) to 4 (all of the time), yielding a total score ranging from 0 to 24. Higher scores indicate greater levels of psychological distress. In this study, maternal psychological distress demonstrated good internal consistency (Cronbach’s α = 0.85).

### Parenting Style

Based on Baumrind’s conceptual framework, parenting practices are commonly characterized along two core dimensions: demandingness and responsiveness (Baumrind, 1971). Although both dimensions are considered important, parental responsiveness, reflected in warmth, support, and sensitivity to children’s needs, is particularly influential for adolescents’ socioemotional development, self-evaluative processes, and mental health outcomes. The China Family Panel Studies (CFPS) includes validated measures that capture the responsiveness dimension of parenting. Thus, the present study assessed parenting practices using a parental responsiveness scale derived from the CFPS. Adolescents reported their perceptions of parental responsiveness based on five items, which assessed behaviors such as encouraging effort, promoting independent thinking, explaining the reasons behind rules, discussing problems, and engaging in open communication. All items were rated on a 5-point Likert scale ranging from 1 (never) to 5 (very often). Item scores were summed to create a total score ranging from 5 to 25, with higher scores indicating higher levels of parental responsiveness. The scale demonstrated acceptable internal consistency in the present sample (Cronbach’s α = 0.72).

#### Self-Esteem

The Rosenberg Self-Esteem Scale (RSES) serves as a key instrument for measuring self-esteem. This scale was initially designed to assess adolescents’ overarching feelings regarding self-worth and self-acceptance (Rosenberg & Pearlin, 1978). The scale consists of 10 items rated on a 5-point Likert scale ranging from 1 (strongly disagree) to 5 (strongly agree), with five items reverse-coded. Sample items include statements such as “I feel that I have a number of good qualities” and “On the whole, I am satisfied with myself.” Item scores were summed to create a total self-esteem score, with higher scores indicating higher levels of self-esteem. In the present study, the RSES demonstrated acceptable internal consistency (Cronbach’s α = 0.71).

### Missing Data

Missing data were inevitable given the 10-year longitudinal design of the CFPS. The baseline sample included 2,245 children who met the age criteria in 2010 (T1). By 2020 (T6), 1,143 participants remained in the survey, corresponding to a retention rate of 50.9% (see Table S1). Rather than excluding participants with incomplete follow-up, all baseline respondents were retained for analysis in order to minimize potential bias associated with case deletion and to maximize statistical power. To assess attrition, participants who completed the 2020 wave were compared with those who were lost to follow-up on baseline demographic characteristics and family SES indicators. As shown in Table S2, no significant differences were observed in age, gender, only-child status, or parental education. Significant differences were found in urban-rural residence, co-residence with parents, household income, and parental occupational status. Effect sizes were calculated using Cramér’s V for categorical variables and Cohen’s d for continuous variables. All effect sizes were small (Cramér’s V and |d| ≤ 0.17), suggesting that although attrition was not completely random, the magnitude of differences between retained and attrited participants was limited. Missing data were handled using full information maximum likelihood (FIML) estimation in subsequent analyses. This approach allows the inclusion of all available cases and produces unbiased parameter estimates under the assumption that data are missing at random (MAR). All structural models were estimated using maximum likelihood methods with robust standard errors.

### Analytical Strategy

All data management and preliminary analyses were conducted using Stata 16.0. Descriptive statistics and bivariate correlations were first examined to assess variable distributions and associations. Subsequent longitudinal analyses were performed using Mplus 8.3 (Muthén & Muthén, 2017).

To model the developmental pattern of adolescent depressive symptoms, latent growth models (LGMs) were estimated. LGMs allow for the examination of intraindividual change over time as well as interindividual differences in developmental trajectories by specifying latent intercept and slope factors based on repeated measures (Berlin et al., 2014). In the present study, depressive symptoms measured in 2016, 2018, and 2020 were used as indicators of the latent growth factors. The intercept factor represented the overall level of depressive symptoms, whereas the slope factor captured the rate of change across adolescence. Linear growth models were specified given the number of measurement occasions and prior empirical evidence.

Building on the fitted growth models, longitudinal mediation models were estimated to examine indirect pathways linking early family SES to adolescent depressive symptom trajectories. Family SES measured at baseline (2010) was specified as the exogenous predictor. Maternal psychological distress and parenting responsiveness assessed in 2014 were modeled as parallel mediators, followed by adolescent self-esteem measured in 2014 as a downstream mediator. The latent intercept and slope of depressive symptoms served as outcome variables. This stepwise modeling strategy was adopted to evaluate theoretically informed pathways while maintaining model parsimony.

Given that family SES is a multidimensional construct, three separate models were estimated using household income, highest parental occupational status, and highest parental educational attainment as focal predictors, respectively. All models controlled for baseline covariates, including gender, age, urban-rural residence, only-child status, and co-residence with parents. Model fit was evaluated using standard indices, including the comparative fit index (CFI), Tucker–Lewis index (TLI), root mean square error of approximation (RMSEA), and standardized root mean square residual (SRMR). Indirect effects were estimated using maximum likelihood methods with robust standard errors.

## Results

### Descriptive Statistics

Descriptive statistics and correlations among variables are presented in Table 1. The results indicate that, across most observation periods, higher family SES at T1 was consistently associated with lower levels of depressive symptoms measured at T4, T5, and T6 (*r* = − 0.05 to − 0.12, *p* < 0.05). Maternal mental health problems assessed at T3 were positively correlated with adolescent depressive symptoms at all subsequent waves (*r* = 0.12 to 0.15, *p* < 0.001), whereas parenting responsiveness and adolescent self-esteem were negatively correlated with depressive symptoms (*r* = − 0.09 to − 0.17, *p* < 0.001). Parenting responsiveness was positively associated with adolescent self-esteem (*r* = 0.13, *p* < 0.001).

**Table 1:** Descriptive statistics and correlations among the study variables (*N* = 2245)

	M	SD	1	2	3	4	5	6	7	8	9	10	11	12	13	14
1.T1 Household income	8.35	0.99	1.00													
2.T1 Parental occupational status	33.21	14.69	0.38^***^	1.00												
3.T1 Parental education level	8.82	3.66	0.39^***^	0.50^***^	1.00											
4.T4 CES-D	11.88	2.99	-0.08^**^	-0.05^*^	-0.08^**^	1.00										
5.T5 CES-D	12.28	3.20	-0.08^**^	-0.06^*^	-0.08^**^	0.26^***^	1.00									
6.T6 CES-D	12.63	3.56	-0.12^***^	-0.06^*^	-0.11^***^	0.21^***^	0.61^***^	1.00								
7.T3 Maternal mental health	33.59	7.51	-0.20^***^	-0.12^***^	-0.17^***^	0.12^***^	0.14^***^	0.15^***^	1.00							
8.T3 Parenting Style	18.24	3.49	0.11^***^	0.10^***^	0.10^***^	-0.13^***^	-0.11^***^	-0.15^***^	-0.05	1.00						
9.T3 Self-esteem	35.84	3.80	0.12^***^	0.11^***^	0.20^***^	-0.15^***^	-0.17^***^	-0.09^***^	-0.08^***^	0.13^***^	1.00					
10.Gender	0.54	0.50	0.00	0.03	-0.01	-0.06^*^	-0.06^*^	-0.04	-0.05	-0.05^*^	-0.01	1.00				
11.Age	6.45	1.12	-0.01	-0.03	-0.01	0.09^***^	0.10^***^	0.05^*^	0.02	0.01	-0.07^**^	-0.01	1.00			
12.Urban	0.37	0.48	0.35^***^	0.37^***^	0.40^***^	-0.07^**^	-0.04	-0.07^**^	-0.19^***^	0.12^***^	0.17^***^	-0.03	-0.04	1.00		
13.Only child	0.23	0.42	0.27^***^	0.22^***^	0.33^***^	-0.08^**^	-0.03	-0.02	-0.16^***^	0.12^***^	0.10^***^	0.07^**^	-0.01	0.26^***^	1.00	
14.Parental co-residence	0.62	0.49	0.08^***^	0.06^**^	0.06^**^	-0.05	-0.03	-0.05	-0.03	0.07^**^	0.07^**^	0.04	-0.07^***^	0.09^***^	0.14^***^	1.00

Note: *p < 0.05, **p < 0.01, ***p < 0.001

### Latent Growth Model

An unconditional latent growth model (LGM) was specified to examine the developmental trajectory of depressive symptoms across three measurement occasions during adolescence. In this model, the intercept factor loadings for the three observed indicators were fixed at 1, and the slope factor loadings were fixed at 0, 1, and 2 to represent T4, T5, and T6, respectively. The model demonstrated excellent fit to the data (χ2/*df* = 254.77, RMSEA = 0.016, CFI = 0.999, TLI = 0.998, SRMR = 0.008). The estimated mean of the intercept was 11.928 (SE = 0.070, *p* < 0.001), indicating the initial level of depressive symptoms. The estimated mean slope was 0.357 (SE = 0.051, *p* < 0.001), suggesting a significant increasing trend in depressive symptoms over the three waves. In addition, the variances of both the intercept (σ2 = 3.077, SE = 0.477, *p* < 0.001) and the slope (σ2 = 2.513, SE = 0.317, *p* < 0.001) were significant, indicating substantial inter-individual variability in both the initial level and rate of change of depressive symptoms. The covariance between the intercept and slope was negative but did not reach statistical significance (*r* = − 0.348, *p* > 0.05).

### The Chain Multiple Mediation Model

Correlation analyses indicated that the associations among household income at T1, maternal mental health and parenting style at T3, adolescent self-esteem at T3, and the intercept and slope of depressive symptom trajectories were appropriate for testing the proposed chain multiple mediation model. Gender, age, urban-rural residence, only-child status, co-residence with parents, and parental occupation and education were included as covariates. The overall model demonstrated acceptable fit to the data (χ2/*df* = 33.63, CFI = 0.99, TLI = 0.98, RMSEA = 0.02, SRMR = 0.02).

**Fig. 1:**
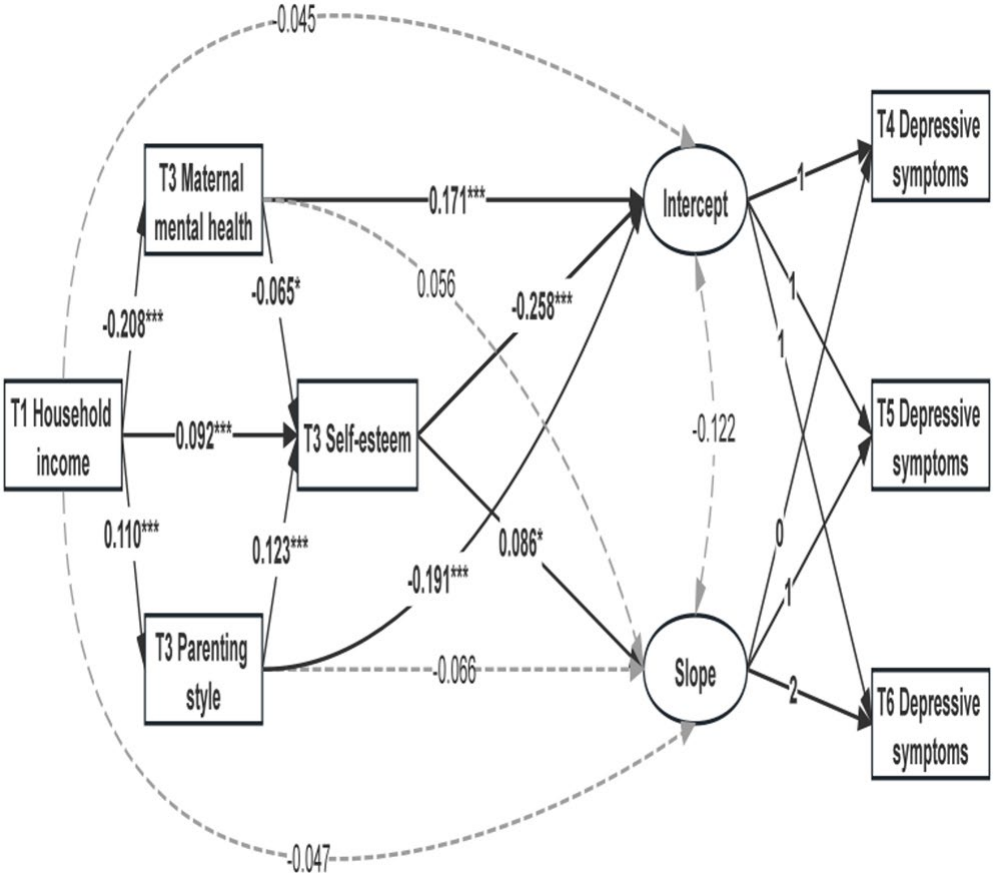
Chain Multiple Mediation Model (Household income, N = 2245). Note: All variables were standardized. *p < 0.05, **p < 0.01, ***p < 0.001

Effects on the intercept of depressive symptoms. As shown in Fig. 1; Table 2, household income at T1 did not directly predict the intercept of adolescent depressive symptoms (β = −0.045, *p* = 0.293). However, the total indirect effect from household income to the intercept was statistically significant (β = −0.087, 95% CI [− 0.121, − 0.059]), indicating a fully indirect association. Specifically, household income was indirectly associated with a lower initial level of depressive symptoms through maternal mental health (INC → MMH → IDEP; β = −0.036, 95% CI [− 0.060, − 0.016]) and parenting style (INC → PS → IDEP; β = −0.021, 95% CI [− 0.038, − 0.009]). In addition, self-esteem independently mediated the association between household income and the intercept of depressive symptoms (INC → SE → IDEP; β = −0.024, 95% CI [− 0.040, − 0.011]).

**Table 2:** The direct and indirect effects of the chain multiple mediation model (Household income)

Effect types	Effect	Boot SE	95% CI	*p*-values
INC to Intercept				
*Direct effect*	−0.045	0.043	[−0.130, 0.038]	0.293
*Total indirect effect*	−0.087	0.016	[−0.121,−0.059]	0.000
INC → MMH → IDEP	−0.036	0.011	[−0.060,−0.016]	0.002
INC → PS→ IDEP	−0.021	0.007	[−0.038,−0.009]	0.005
INC → SE → IDEP	−0.024	0.007	[−0.040,−0.011]	0.001
INC → MMH→ SE → IDEP	−0.004	0.002	[−0.008,−0.001]	0.041
INC → PS → SE → IDEP	−0.003	0.001	[−0.007,−0.002]	0.008
INC to Slope				
*Direct effect*	−0.047	0.033	[−0.144, 0.020]	0.162
*Total indirect effect*	−0.008	0.009	[−0.028, 0.010]	0.373
INC → MMH→ SDEP	−0.012	0.008	[−0.028, 0.003]	0.143
INC → PS→ SDEP	−0.007	0.004	[−0.018, 0.000]	0.101
INC → SE → SDEP	0.008	0.004	[0.002, 0.017]	0.041
INC → MMH → SE → SDEP	0.001	0.001	[0.000, 0.003]	0.126
INC → PS → SE → SDEP	0.001	0.001	[0.000, 0.003]	0.065

Note: *INC* Household income; *MMH* maternal mental health; *IDEP* Initial level of adolescent depressive symptoms; *PS* Parenting Style; *SE* self-esteem; *SDEP* Rate of progression of adolescent depressive symptoms

Moreover, two chain multiple mediation pathways were supported. Higher household income was associated with better maternal mental health, which in turn predicted higher adolescent self-esteem and subsequently a lower intercept of depressive symptoms (INC → MMH → SE → IDEP; β = −0.004, 95% CI [− 0.008, − 0.001]). A similar chain effect was observed via parenting style and self-esteem (INC → PS → SE → IDEP; β = −0.003, 95% CI [− 0.007, − 0.002]). Together, these findings suggest that household income influences adolescents’ initial depressive symptom levels primarily through both parallel and sequential pathways involving family processes and self-esteem.

Effects on the slope of depressive symptoms. Regarding the slope of depressive symptoms, the direct effect of household income was not statistically significant (β = −0.047, *p* = 0.162), nor was the total indirect effect (β = −0.008, 95% CI [− 0.028, 0.010]). Among the specific indirect paths, only self-esteem showed a significant mediating effect, such that higher household income predicted higher self-esteem, which was associated with a steeper increase in depressive symptoms over time (INC → SE → SDEP; β = 0.008, 95% CI [0.002, 0.017]). Indirect effects through maternal mental health, parenting style, and the chain pathways were not statistically significant.

**Fig. 2:**
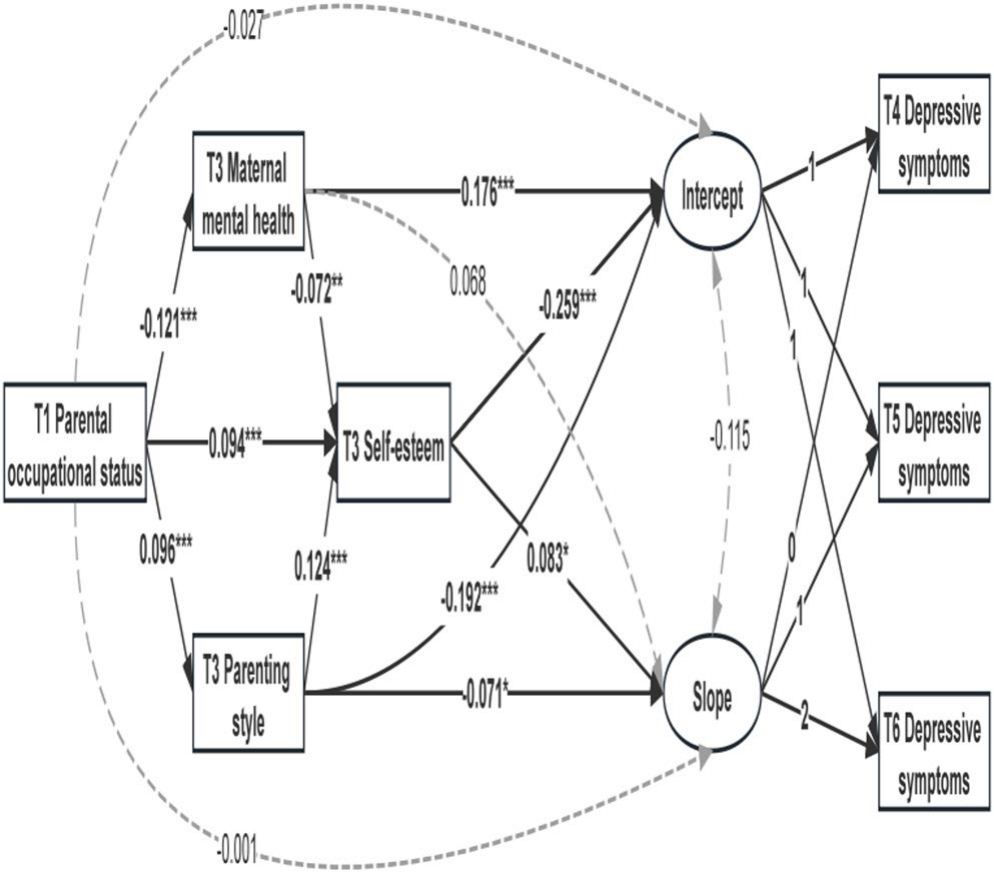
Chain Multiple Mediation Model (parental occupational status, *N* = 2245). Note: All variables were standardized. *p < 0.05, **p < 0.01, ***p < 0.001

Supplementary analyses using parental occupational status and education level. To examine the robustness of the findings across different indicators of family SES, the same chain multiple mediation model was further estimated using parents’ highest educational attainment and parents’ highest occupational status (ISEI) as alternative predictors. The model fit was excellent for both parental education (χ2/*df* = 33.93) and parental occupational status (χ2/*df* = 31.96), with consistent values across other indices (CFI = 0.99, TLI = 0.98, RMSEA = 0.02, SRMR = 0.02). Overall, the results based on parental education were highly consistent with those obtained for household income, showing similar patterns in both the intercept and slope of adolescent depressive symptom trajectories (see Fig. S1 and Table S3). In contrast, the model using parents’ highest occupational status revealed a partially distinct pattern, particularly with respect to the slope of depressive symptoms. As shown in Fig. 2; Table 3, the direct effect of ISEI on the slope was not statistically significant (β = −0.001, *p* = 0.985), nor was the total indirect effect (β = −0.006, 95% CI [− 0.019, 0.008]). However, a specific indirect pathway through parenting style was supported by the bootstrap confidence interval. Higher parental occupational status was associated with more favorable parenting practices, which in turn predicted a slower increase in depressive symptoms over time (ISEI → PS → SDEP; β = −0.007, 95% CI [− 0.017, − 0.001]). In addition, self-esteem also shaped the slope of depressive symptoms. Consistent with the household income model, parental occupational status was positively associated with adolescent self-esteem, which was further linked to a steeper increase in depressive symptoms over time (ISEI → SE → SDEP; β = 0.008, 95% CI [0.002, 0.018]). Indirect effects through maternal mental health alone, as well as the chain pathways involving maternal mental health or parenting style and self-esteem, were not statistically significant.

**Table 3:** The direct and indirect effects of the chain multiple mediation model (Parental occupational status)

Effect types	Effect	Boot SE	95% CI	*p*-values
ISEI to Intercept				
*Direct effect*	−0.027	0.043	[−0.019, 0.059]	0.528
*Total indirect effect*	−0.069	0.013	[−0.097,−0.047]	0.000
ISEI → MMH → IDEP	−0.021	0.007	[−0.038,−0.009]	0.003
ISEI → PS→ IDEP	−0.018	0.007	[−0.035,−0.008]	0.007
ISEI → SE → IDEP	−0.024	0.008	[−0.042,−0.012]	0.001
ISEI → MMH→ SE → IDEP	−0.002	0.001	[−0.005,−0.001]	0.038
ISEI → PS → SE → IDEP	−0.003	0.001	[−0.006,−0.001]	0.010
ISEI to Slope				
*Direct effect*	−0.001	0.033	[−0.066, 0.067]	0.985
*Total indirect effect*	−0.006	0.007	[−0.019, 0.008]	0.426
ISEI → MMH→ SDEP	−0.008	0.005	[−0.019, 0.000]	0.090
ISEI → PS→ SDEP	−0.007	0.004	[−0.017,−0.001]	0.085
ISEI → SE → SDEP	0.008	0.004	[0.002, 0.018]	0.051
ISEI → MMH → SE → SDEP	0.001	0.000	[0.000, 0.002]	0.132
ISEI → PS → SE → SDEP	0.001	0.001	[0.000, 0.003]	0.078

Note: *ISEI* parental occupational status; *MMH* maternal mental health; *IDEP* Initial level of adolescent depressive symptoms; *PS* Parenting Style; *SE* self-esteem; *SDEP* Rate of progression of adolescent depressive symptoms

In summary, although different indicators of family SES generally operate through similar family and individual mechanisms, parental occupational status may exert a unique influence on the developmental pace of depressive symptoms through parenting practices, highlighting the importance of distinguishing between structural and relational components of socioeconomic resources.

## Discussion

Empirical and theoretical work has consistently linked lower family SES with elevated depressive symptoms in adolescence (Zeng & Xu, 2024). Although tracking depressive trajectories provides valuable insight into the dynamic course of symptom development, the specific pathways through which early SES influences these trajectories, particularly those integrating both family processes and self-system mechanisms, remain underexplored. The present study extends prior research by (a) employing a multidimensional assessment of family SES that includes household income, parental education, and occupational status; (b) testing a sequential mediation model that incorporates maternal mental health, parenting style, and adolescent self-esteem; and (c) applying a longitudinal multi-wave design to differentiate between initial symptom levels (intercept) and their rate of change over time (slope). Partially supporting the hypotheses, maternal mental health and parenting style mediated the effects of both household income and parental education on the intercept of depressive symptoms. Adolescent self-esteem further operated as a downstream mediator in these pathways. In addition, parenting style and self-esteem mediated the association between parental occupational status and the slope of depressive symptoms. A notable and consistent pattern emerged across all SES indicators: higher self-esteem predicted a steeper increase in depressive symptoms over time, a finding that diverges from traditional vulnerability models (Orth et al., 2008). This aligns with recent work suggesting that certain intrapersonal resources may interact with developmental demands in complex ways, potentially intensifying emotional reactivity during adolescence (Chiang & Bai, 2024). Thus, while self-esteem may confer protection against initial depression levels, it may also coincide with heightened awareness of or responsiveness to developmental stressors, thereby accelerating symptom progression across adolescence. These results provide clearer evidence on how different dimensions of family SES shape depressive trajectories via separate family and psychological pathways.

### The Direct Link between Family SES and Adolescents’ Depressive Trajectories

In contrast to prior studies that have documented direct associations between family SES and adolescent depressive symptoms (Zhou et al., 2018), the present study found that family SES was no longer directly associated with either the intercept or the slope of depressive symptom trajectories after accounting for parental mental health, parenting practices, and adolescent self-esteem. This pattern indicates that the influence of family SES on depressive symptom trajectories is fully mediated by more proximal family and individual processes. These findings are consistent with the view that socioeconomic disadvantage does not directly affect adolescent mental health but instead operates through the erosion of psychosocial resources within the family context (Kirkbride et al., 2024). From this perspective, SES functions as a distal structural condition whose effects are transmitted through everyday family processes (Conger & Donnellan, 2007). Thus, the disappearance of the direct SES effect after including the mediators supports a full mediation model and confirms the importance of examining how socioeconomic conditions shape adolescents’ lived experiences. By modeling depressive symptoms as longitudinal trajectories, the present study further demonstrates that the long-term course of depression is shaped less by socioeconomic background alone and more by the family processes that socioeconomic conditions set in motion (Conger et al., 2010).

### The Influence of Family SES on Maternal Mental Health and Parenting Style

The present findings further support the view that family SES is closely linked to maternal psychological functioning and parenting practices. Lower family SES was associated with poorer maternal mental health and less adaptive parenting practices, consistent with the Family Stress Model (Leivas et al., 2018). Socioeconomic strain is often accompanied by chronic stressors such as economic insecurity, limited access to resources, and role overload, which may disproportionately affect mothers who typically assume primary caregiving responsibilities (Scrimin et al., 2022). These parent-level processes, in turn, were associated with adolescents’ depressive outcomes, corroborating prior longitudinal evidence showing that maternal depression and suboptimal parenting practices predict higher levels and more rapid increases in adolescent depressive symptoms over time (Wang et al., 2018). Importantly, the mother-child relationship is a central context for children’s and adolescents’ emotional development and emotion regulation (Hollenstein et al., 2017). Within the family dynamic system, mothers often function as key emotional transmitters, and their psychological states exert pronounced spillover effects on family interactions. Previous research has shown that maternal depression and anxiety mediate the association between SES and parenting behaviors (Newland et al., 2013). The present study found that both maternal mental health and parenting practices mediated the association between family SES and adolescents’ depressive symptom trajectories, showing that parental functioning as a key mechanism through which socioeconomic disadvantage is translated into emotional risk for adolescents. This finding aligns with prior studies demonstrating that parenting behaviors partially explain differences in adolescents’ mental health (Williams et al., 2024).

### The Mediating Role of Self-Esteem

Self-esteem played a significant mediating role in the association between family SES and adolescents’ depressive symptom trajectories. Lower family SES was associated with lower levels of self-esteem, which in turn predicted higher initial levels of depressive symptoms. This finding is consistent with prior research suggesting that socioeconomic disadvantage may constrain opportunities for positive self-evaluation and perceived control, thereby undermining self-worth (Twenge & Nolen-Hoeksema, 2002). By embedding self-esteem within a developmental trajectory framework, the present longitudinal design extends the existing literature by demonstrating that self-esteem not only relates to concurrent depressive symptoms but also helps explain why adolescents from lower socioeconomic backgrounds have higher initial levels of depression. These results support the vulnerability model of self-esteem, which conceptualizes low self-esteem as a stable risk factor that increases susceptibility to depression (Sowislo & Orth, 2013). Notably, self-esteem also demonstrated a significant indirect effect on the slope of depressive symptoms, but this association was positive, indicating that adolescents with higher self-esteem showed a more rapid increase in depressive symptoms over time. Although seemingly counterintuitive, this pattern highlights the importance of distinguishing between initial symptom levels and developmental change. One possible explanation is that this finding reflects heightened self-consciousness and evaluative sensitivity that are characteristic of adolescence (Pfeifer & Berkman, 2018). As social comparison intensifies and academic, social, and identity-related challenges accumulate, adolescents with higher self-esteem may experience greater emotional vulnerability during later stages of adolescence (Schöne et al., 2015). Early adolescent self-esteem may be accompanied by elevated self-expectations or more conditional forms of self-worth, rendering individuals particularly sensitive to perceived failure or discrepancies between ideal and actual selves (Ferguson et al., 2010). As developmental demands increase, these adolescents may begin with lower levels of depressive symptoms, yet exhibit steeper increases over time. This interpretation points to the dynamic and context-dependent nature of self-esteem and cautions against viewing it as a uniformly protective factor across developmental stages. Future research should examine whether this pattern reflects a developmental disappointment effect or whether it is moderated by contextual supports and environmental resources.

### The Chain-multiple-mediating Role of Maternal Mental Health, Parenting Style, and Self-Esteem

Beyond their independent mediating roles, maternal mental health, parenting practices, and adolescent self-esteem formed a serial mediation pathway linking family SES to adolescents’ depressive symptom trajectories. Specifically, family SES influenced maternal psychological functioning and parenting practices, which in turn shaped adolescents’ self-esteem and ultimately affected both the initial level and the rate of change of depressive symptoms. This sequential process shows how distal socioeconomic conditions become embedded in adolescents’ internal psychological functioning through successive family-level and individual-level mechanisms (Zietz et al., 2022). These findings are consistent with prior research demonstrating that supportive parenting and parental psychological well-being provide a critical foundation for the development of adolescents’ self-worth (Barber et al., 2005), and they extend previous studies that have examined these constructs primarily as parallel mediators rather than as part of a temporally ordered process (Xie & Liu, 2026). Family income was associated with adolescents’ internalizing problems through parental depressive symptoms and parenting behaviors, supporting the plausibility of each link in the proposed sequence (Bøe et al., 2014). By integrating these mechanisms within a serial mediation framework and linking them to depressive symptom trajectories rather than cross-sectional outcomes, the present study consolidates and extends existing evidence.

Importantly, the salience of this family-centered pathway may be especially strong in the Chinese cultural context, where families play a central role in adolescents’ emotional socialization and self-evaluative development (Shi et al., 2024). In contrast to more individualistic contexts, Chinese adolescents tend to rely more heavily on parental support, feedback, and expectations as primary sources of self-worth, especially under conditions of socioeconomic constraint (Xu et al., 2019). Consequently, parental psychological distress and parenting responsiveness may exert a stronger influence on adolescents’ self-esteem, thereby amplifying their downstream associations with depressive symptom trajectories (Cui et al., 2025). Moreover, cultural emphases on educational achievement and upward mobility may intensify parents’ emotional involvement and regulatory practices when family resources are limited (Wang et al., 2024), potentially increasing psychological pressure on adolescents and undermining their self-evaluative processes (Xu et al., 2025).

Differences across SES indicators further enrich this interpretation. While family income and parental education had broadly similar mediation patterns, parental occupational status showed distinct associations with changes in depressive symptoms over time. Occupational status may capture enduring social prestige and perceived social standing, which are especially important in societies characterized by strong social comparison and status consciousness, such as China (Landstedt et al., 2016). These perceptions may shape adolescents’ expectations regarding future mobility and social positioning, thereby influencing the rate of change in depressive symptoms. This distinction highlights the importance of conceptualizing SES as a multidimensional construct rather than as a single composite indicator (Boer et al., 2024).

Although family cultural values and broader social support networks were not directly measured, the present findings suggest that their influence may be indirectly embedded within the observed family processes. Prior research indicates that when external institutional or community-based support is limited, family-level psychological and relational processes become the primary channels through which socioeconomic conditions shape adolescent mental health (Chen, 2025). Future studies incorporating explicit measures of cultural values and social support would help to further clarify how these contextual factors interact with family processes to influence adolescents’ emotional development.

### Limitations and Future Directions

Several limitations should be acknowledged. First, sample attrition over the extended follow-up period may limit generalizability, although baseline differences between retained and attrited participants were modest and missing data were addressed using full information maximum likelihood estimation. Second, although the study covered a relatively long developmental period, additional measurement occasions and follow-up into early adulthood would further strengthen conclusions regarding long-term depressive symptom trajectories. Third, the present analyses focused primarily on family-level processes. Future research should examine more complex and dynamic mechanisms, including potential moderating roles of gender, peer relationships, and school contexts. Despite these limitations, this study advances understanding of how family SES shapes adolescent depressive symptom trajectories through interconnected family and individual processes.

## Conclusion

Although prior studies have documented associations between family socioeconomic status and adolescent depressive symptoms, little was known about how distinct dimensions of SES shape the longitudinal development of depressive symptoms through interconnected family and individual mechanisms. Guided by an extended family stress model, this study used longitudinal data and latent growth modeling to examine these pathways. Lower family SES was associated with poorer maternal mental health and less adaptive parenting practices, which in turn predicted lower adolescent self-esteem and more unfavorable depressive trajectories. Family income and parental education exhibited largely similar mediation patterns, whereas parental occupational status showed a distinct association with changes in depressive symptoms over time, highlighting the need to treat family SES as a multidimensional construct. These findings advance developmental research by demonstrating that socioeconomic influences on adolescent emotional development are embedded in proximal family dynamics and individual psychological resources that shape depressive trajectories over time.

## Supplementary Information

Below is the link to the electronic supplementary material.

Supplementary Material 1

## Data Availability

The datasets supporting the conclusions of this article are available in the CFPS repository, http://www.isss.pku.edu.cn/cfps/en/documentation/questionnaires/index.htm? CSRFT=YPAC-8N0M-L215-I0D4-BP4X-GPXD-IPHR-KM6Y. And the datasets can be obtained after sending a data user agreement to the CFPS team, http://www.isss.pku.edu.cn/cfps/download/index#/ fileTreeList.
