# Supplementary material for: Longitudinal Associations Between Family Socioeconomic Status and Adolescent Depressive Symptom Trajectories in China: A Chain Multiple Mediation Model
Source: J Youth Adolesc. 2026 Apr 7;55(7):1804–18. doi: 10.1007/s10964-026-02355-4 (PMC13328128; doi:10.1007/s10964-026-02355-4)
Supplement: Supplementary file 1 — Supplementary Material 1 [file 10964_2026_2355_MOESM1_ESM.docx]

**Appendix:**

**(1) Sample Attrition**

At baseline in 2010, the sample consisted of 2,245 eligible participants based on age criteria. By 2020, after excluding cases lost due to refusal to respond or other reasons, 1,143 participants were retained, resulting in a retention rate of 50.9%. The annual sample attrition is presented in Table S1.

**Table S1** Sample Attrition Across Survey Waves

| Wave | Number of participants who participated in both 2010 and 2020 surveys (*N*) |
| --- | --- |
| 2010 | 2245 |
| 2012 | 1087 |
| 2014 | 1060 |
| 2016 | 981 |
| 2018 | 1122 |
| 2020 | 1143 |

**（2）Comparison of Characteristics Between Retained and Attrited Samples**

This study further conducted difference tests on the basic characteristics and family socioeconomic status variables between the retained sample and the attrited sample. The results in Table S2 show that there were no significant differences between the retained and attrited samples in terms of age, gender, only-child status, or parental education level. However, differences were observed in urban-rural distribution, co-residence with parents, household income, and parental occupational status. To assess the magnitude of these differences, effect sizes were further calculated: Cramér's V for categorical variables and Cohen's d for continuous variables. A Cramér's V of 0.10 indicates a small effect, 0.30 a medium effect, and 0.50 a large effect. The results of this study show that all effect sizes were below 0.17, indicating that the actual differences between the retained and attrited samples were small.

**Table S2** Comparison of Basic Characteristics and Family Socioeconomic Status Between the Retained and Attrited Samples in 2010

| **Variable** | **Retained Sample**  (N = 1143) | **Attrited Sample**  (N = 1102) | **Test Statistic**  **$\chi^{2}$/$t$** | **Effect Size** |
| --- | --- | --- | --- | --- |
| Age, Mean (SD) | 6.46(1.13) | 6.44(1.11) | 0.332 | 0.014 |
| Gender, N(%) |  |  | 0.598 | 0.016 |
| Male | 611(50.16) | 607(49.84) |  |  |
| Female | 532(51.80) | 495(48.20) |  |  |
| Urban-Rural, N(%) |  |  | 27.182*** | 0.110 |
| Urban | 360(43.69) | 464(56.31) |  |  |
| Rural | 783(55.10) | 638(44.90) |  |  |
| Only Child, N(%) |  |  | 0.455 | 0.014 |
| Yes | 272(52.21) | 249(47.79) |  |  |
| No | 871(50.52) | 853(49.48) |  |  |
| Co-residence with Parents, N(%) |  |  | 64.372*** | 0.169 |
| Yes | 799(57.91) | 589(42.44) |  |  |
| No | 344(40.14) | 513(59.86) |  |  |
| Household Income, Mean (SD) | 8.27(0.97) | 8.42(1.01) | 3.560*** | 0.150 |
| Parental Occupational Status, Mean (SD) | 32.51(14.40) | 33.94(14.95) | 2.305** | 0.097 |
| Parental Education Level, Mean (SD) | 8.75(3.40) | 8.94(3.29) | 1.349 | 0.057 |

Note: p-values are derived from t-tests and chi-square tests. *p < 0.05，**p < 0.01，***p <0.001.

**（3）Mediation Models Using Parental Education Level and Adolescent Depressive Symptom Trajectories**

Supplementary Analysis Based on Parental Education Level. To examine the robustness of the findings across different indicators of family SES, we further estimated the same chain multiple mediation model using parents' highest educational attainment as an alternative predictor. The parental education model demonstrated excellent fit (χ²/df = 33.93, CFI = 0.99, TLI = 0.98, RMSEA = 0.02, SRMR = 0.02). Overall, the results based on parental education were highly consistent with those from the household income model, showing similar patterns for both the intercept and slope of the adolescent depressive symptom trajectories (see Fig. S1 and Table S3).


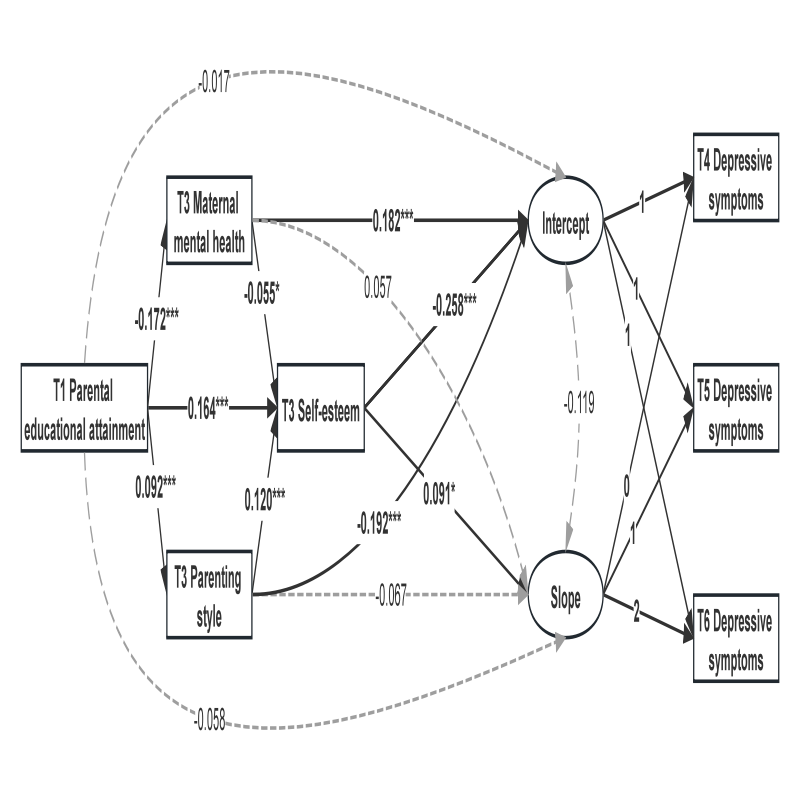


**Fig. S1** Chain Multiple Mediation Model (Parental Education Level, *N* = 2245)

Note: All variables were standardized. **p* < 0.05, ***p* < 0.01, ****p* < 0.001

.

**Table S3** Direct and Indirect Effects of the Chain Multiple Mediation Model (Parental Education Level)

| **Effect types** | **Effect** | **Boot SE** | **95% CI** | **p-values** |
| --- | --- | --- | --- | --- |
| EDU to Intercept |  |  |  |  |
| *Direct effect* | -0.017 | 0.042 | [-0.102,0.065] | 0.683 |
| *Total indirect effect* | **-0.096***** | 0.016 | [-0.131,-0.069] | 0.000 |
| EDU → MMH → IDEP | **-0.031**** | 0.010 | [-0.053,-0.014] | 0.002 |
| EDU → PS→ IDEP | **-0.018**** | 0.006 | [-0.033,-0.008] | 0.005 |
| EDU → SE → IDEP | **-0.042***** | 0.010 | [-0.064,-0.025] | 0.000 |
| EDU → MMH→ SE → IDEP | -0.002 | 0.001 | [-0.006,0.000] | 0.084 |
| EDU → PS → SE → IDEP | **-0.003*** | 0.001 | [-0.006,-0.001] | 0.013 |
|  |  |  |  |  |
| EDU to Slope |  |  |  |  |
| *Direct effect* | -0.058 | 0.035 | [-0.126,0.010] | 0.102 |
| *Total indirect effect* | 0.001 | 0.010 | [-0.019,0.020] | 0.936 |
| EDU → MMH→ SDEP | -0.010 | 0.007 | [-0.024,0.002] | 0.141 |
| EDU → PS→ SDEP | -0.006 | 0.004 | [-0.016,0.000] | 0.109 |
| EDU → SE → SDEP | **0.015*** | 0.006 | [0.004,0.029] | 0.019 |
| EDU → MMH → SE → SDEP | 0.001 | 0.001 | [0.000,0.003] | 0.167 |
| EDU → PS → SE → SDEP | 0.001 | 0.001 | [0.000,0.003] | 0.068 |

Note: *EDU* parental education level; *MMH* maternal mental health; *IDEP* Initial level of adolescent depressive symptoms; *PS* Parenting Style; *SE* self-esteem; *SDEP* Rate of progression of adolescent depressive symptoms. **p* < 0.05, ***p* < 0.01, ****p* < 0.001.
